# Association between oral health and frailty among older cancer patients: a cross-sectional study

**DOI:** 10.1007/s00520-026-10650-w

**Published:** 2026-04-20

**Authors:** Xueyan Cheng, Pui Hing Chau, Denise Shuk Ting Cheung

**Affiliations:** School of Nursing, Li Ka Shing Faculty of Medicine, The University of Hong Kong, 5/F, Academic Building, 3 Sassoon Road, Pokfulam, Hong Kong, China

**Keywords:** Oral health, Frailty, Older cancer patients, The United States

## Abstract

**Purpose:**

The association between oral health and frailty in older cancer patients has not been examined. This study aimed to evaluate the associations between oral health and frailty among older cancer patients based on the National Health and Nutrition Examination Surveys (NHANES) 2021–2023.

**Methods:**

615 eligible cancer patients aged ≥ 60 years from the cross-sectional study NHANES 2021–2023. Frailty was measured using a 38-item frailty index. Oral health was measured using a seven-item questionnaire provided by NHANES. Weighted logistic regression models were used to evaluate the association between oral health and frailty. Subgroup analysis was used to examine the difference in the association between the oral health predictors and frailty across covariates groups.

**Results:**

The weighted proportion of older cancer patients with frailty was 47.8%. Worse overall oral health was significantly associated with frailty (OR = 1.13, 95%CI: 1.07, 1.19). Specifically, frailty risk was higher among older cancer patients with worse health in teeth and gums, frequent oral pain, oral-related depression, dietary restrictions, oral-related eating limitations, social embarrassment, and working restrictions (ORs ranging from 1.82 to 5.00). Subgroup analysis identified that males, single/divorced/widowed individuals, those with lower education levels, and smokers showed an elevated risk of frailty relative to specific oral health predictors.

**Conclusion:**

This study demonstrated the association between poor oral health and frailty among older cancer patients. Further longitudinal and interventional studies are warranted to explore the potential utility of routine oral health assessments in identifying frailty risk and informing targeted management strategies in older cancer patients.

**Supplementary Information:**

The online version contains supplementary material available at 10.1007/s00520-026-10650-w.

## Introduction

Frailty is characterized by an age-related decline across multiple physiological system, leading to a diminished ability to maintain homeostasis and an increased vulnerability to adverse health outcomes [1, 2]. The overall prevalence of frailty among older adults worldwide ranges from 10.1% to 38.7% [3, 4]. Notably, among older cancer patients, the prevalence of frailty is higher, reaching approximately 43% [5], likely attributable to the cumulative physiological burden of cancer and its treatments, which exacerbate functional decline and physiological deterioration. Frailty in older cancer survivors correlates with adverse outcomes including mortality, fall, and postoperative complications [5], imposing substantial burdens on health systems through increased service utilization, complex management needs, and higher expenditure [6]. Consequently, identifying predictors of frailty in older cancer patients is critical for developing tailored interventions.

Oral health issues are highly prevalent in older adults, and they have gained recognition as important determinants of overall health and functional status [7]. Emerging evidence suggests that oral health is a crucial, yet underrecognized, factor influencing frailty. Several reviews identified poor oral health—characterized by periodontal disease, tooth loss, and mucosal lesions—as a predictor of frailty in general older adults[8, 9, 40]. The mechanisms underpinning this association are multifaceted; poor oral health can lead to inadequate nutritional intake due to chewing difficulties and altered taste, while chronic oral infections can promote systemic inflammation—both key pathways implicated in the development of frailty [10].

In the context of older cancer survivors, the side effects of cancer treatments—such as chemotherapy, radiotherapy, and surgeries—further exacerbate oral health problems. For instance, studies have reported higher rates of xerostomia (dry mouth) in older cancer patients than in those without cancer (77% [11] vs. 27.2% [12]), altered perception of taste (64.2% [13] vs. 11.9% [14]), dysphasia (54% [15] vs. 33% [16]). Cancer treatment-related toxicities, such as oral mucositis, xerostomia, and dental decay, may compound pre-existing oral health issues, potentially accelerating frailty progression. Additionally, an elevated risk of malnutrition [17] and heightened inflammatory status [18] may further amplify the nutritional and biological pathways linking oral health to frailty in older cancer patients. Despite this plausible link, few studies have examined the association between oral health status and frailty within this vulnerable group.

To our knowledge, no studies have examined the association between oral health and frailty in older cancer patients. To address this gap, we leveraged data from the National Health and Nutrition Examination Surveys (NHANES) 2021–2023 to investigate the association between oral health and frailty among older cancer patients.

## Methods

### Study design and participants

**Fig. 1:**
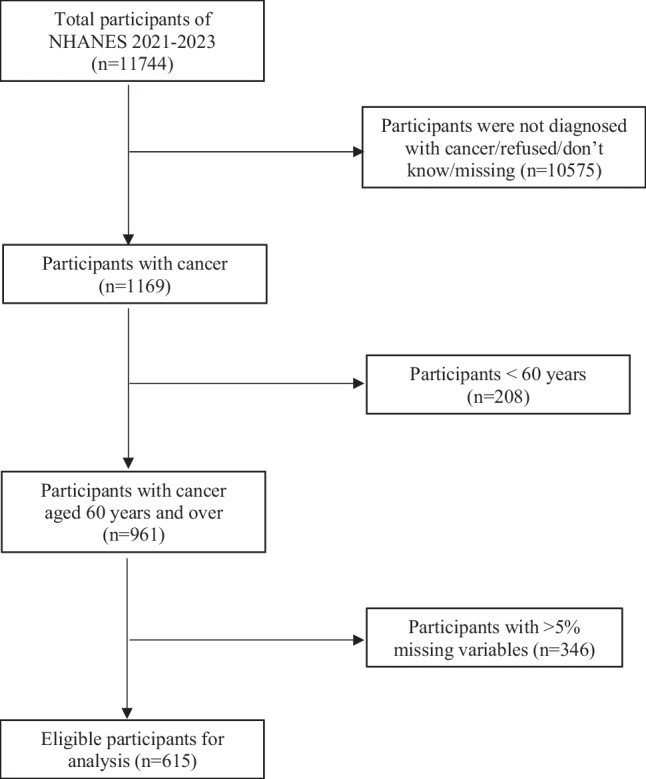
Selection process for eligible participants

This cross-sectional study included a nationally representative survey of Americans (NHANES 2021–2023). NHANES is administered by the Centers for Disease Control and Prevention (CDC) and the National Center for Health Statistics (NCHS) [19]. The NHANES data were collected from the US civilian noninstitutionalized population using a stratified, multistage sampling strategy, which includes publicly available cross-sectional surveys that focus on health and nutritional status. The comprehensive sampling strategies and data collection techniques could be found on the official CDC website at https://www.cdc.gov/nchs/nhanes/. Every participant provided informed consent before the investigation. The eligible older cancer patients in the dataset of NHANES 2021–2023 were those: (1) aged 60 years and over, (2) diagnosed with a confirmed cancer, and (3) with less than 5% missing data for all included variables. The selection process was shown in Fig. 1.

### Frailty Index

The Frailty Index (FI) used in this study includes 38 items and was modified from the previously validated FI in NHANES [20–22] (Supplementary Table S1), including self-reported health, functions, and vital signs, and a laboratory test. The FI was calculated by dividing the number of individual deficits by the total number of possible deficits. The score of FI ranged between 0 and 1, and a higher score was indicative of higher frailty. Based on previous studies using FI in NHANES data, a FI score ≤ 0.21 was “non-frail” and a score of > 0.21 was “frail” [22].

### Oral health

An oral health questionnaire (7 items, score range 1–5) provided by the NHANES was used to estimate self-reported oral health status, with a higher score indicating poorer oral health. Parameters included health of teeth and gums, oral pain, oral-related depression, dietary restrictions, oral-related eating limitations, oral-related social embarrassment, and oral-related working restrictions. For the item on health of teeth and gums, responses were coded as 1 = excellent, 2 = very good, 3 = good, 4 = fair, 5 = poor. For the remaining six adverse items, response options were coded as follows: 1 = never, 2 = hardly ever, 3 = occasionally, 4 = fairly often, 5 = very often (Supplementary Table S2). The total oral health score was calculated by summing the scores across all seven items.

### Other variables

Several individual characteristics potentially associated with frailty were extracted [8–10]. Background characteristics included age (year); gender (male vs. female), and ethnicity (non-Hispanic white vs. others), education levels (≤ high school vs. some college/college), marriage status (married/living with partner vs. unmarried/divorced/widowed), and cancer type (breast/prostate cancer vs. others). Lifestyle-related factors including smoking status (smoked at least 100 cigarettes in total: yes vs. no) and energy and nutrient intake were measured using a 26-item nutrition index for the NHANES cycle-specific USDA Food & Nutrient Database for Dietary Studies [23], and previous studies [20, 24], with higher scores representing worse nutritional status (Supplementary Table S3). Other lifestyle factors, such as drinking status and physical activity, were excluded from the analysis due to substantial missing data.

### Statistical analysis

Statistical analyses were performed with Stata 15.0. Descriptive statistics of the sample characteristics were calculated and stratified by frailty status, with between-group differences tested by t-test and chi-square as appropriate. All percentages and means were weighted using the 2-year sampling weights constructed from the sampling weights provided by NHANES for the general US population-based estimates. Prior to regression modeling, multicollinearity was evaluated using variance inflation factors, with a threshold of variance inflation factors (VIF) < 5 indicating acceptable levels of collinearity [25]. Logistic regression models were used to estimate the association between frailty and oral health, and the adjusted odds ratios (ORs) and 95%CIs were presented. Frailty status (non-frail vs. frail) was the dependent variable, and oral health parameters were independent variables. Two models were constructed: the first model was unadjusted; the second model was adjusted for potential covariates (age, gender, ethnicity, marital status, education, cancer type, smoking status, and nutritional status). We used listwise deletion in the regression analyses. Statistical significance was considered as *p* < 0.05, and all reported probability tests were two-sided. Pre-specified subgroup analyses were conducted to assess any differences in the associations across key baseline characteristics, including gender (male vs. female), age group (< = 70 vs. > 70), marriage status (married/living with partner vs. singled/divorced/widowed), education level (< = high school vs. some college/college), ethnicity (non-Hispanic white vs. others), smoking status (yes vs. no), and nutritional intake level (< = mean score vs. > mean score). Interaction between the oral health parameters and pre-specified subgroups was tested by incorporating their product terms into the regression models. Sensitivity analysis was performed using multiple imputation to handle missing data of both the outcomes and covariates.

### Ethical consideration

The protocols of NHANES were approved by the institutional review board of the National Center for Health Statistics, CDC.

## Results

### Participant characteristics

**Fig. 2:**
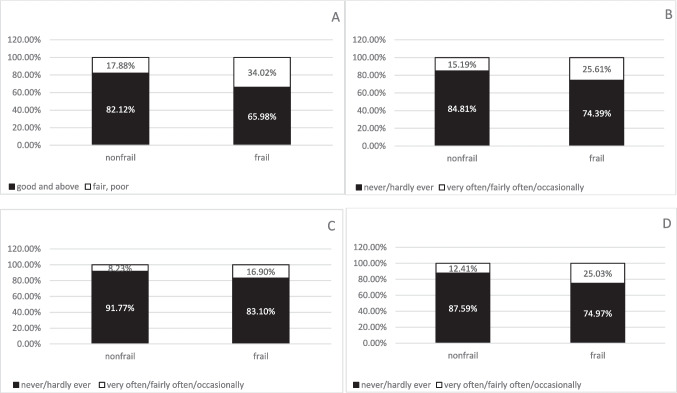

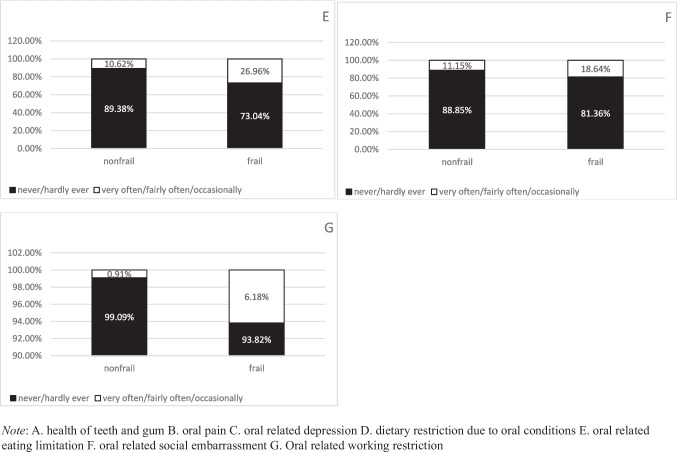
Weighted proportion of oral health prarameters in frail groups

A total of 615 older cancer patients were included in this study. The weighted proportion of older cancer patients with frailty was 47.8% (95%CI: 43.4%, 52.3%). The overall characteristics of the study population are shown in Table 1. Significant differences were observed between the non-frail and frail groups regarding age, marital status, smoking status, and nutritional intake. Compared to non-frail patients, frail patients were significantly older (mean ± SD: 73.2 ± 6.3 vs. 69.5 ± 5.8 years; *p* < 0.001), less likely to be married/living with a partner (50.6% vs. 67.1%; *p* < 0.001), less educated (56.3% vs. 77.2%; *p* < 0.001), more likely to smoke (55.5% vs. 38.8%; *p* < 0.001), and had worse nutritional intakes (0.6 ± 0.2 vs. 0.5 ± 0.2; *p* = 0.026). The weighted proportion of older cancer patients with different oral health parameters were 25.6% (95%CI: 21.9%, 29.6%) for fair/poor health in teeth and gum, 20.2% (95% CI: 16.75%, 24.1%) for oral pain, 12.4% (95%CI: 9.7%, 15.7%) for oral-related depression, 18.4% (95%CI: 15.2%, 22.2%) for dietary restrictions, 18.4% (95%CI: 15.2%, 22.1%) for oral-related eating limitations, 14.7% (95%CI:11.9%, 18.1%) for, and working restrictions 3.4% (95%CI: 0.2%, 5.8%) for social embarrassment, respectively. Weighted proportions of oral health problems stratified by frailty status are presented in Fig. 2.

**Table 1:** Overall characteristics of the study participants by frailty, NHANES 2021–2023

	Non-frail (N = 324)	Frail (N = 291)	Overall (n = 615)	P value
Age, year, mean ± SD	69.50 ± 5.80	73.09 ± 6.29	71.21 ± 6.30	< 0.001
Gender				0.262
Male	142 (46.6%)	130 (41.5%)	272 (44.2%)	
Female	182 (53.4%)	161 (58.5%)	343 (55.8%)	
Marriage				< 0.001
Married/Living with partner	208 (67.1%)	144 (50.6%)	352 (59.2%)	
Unmarried/divorced/widowed	116 (32.9%)	147 (49.4%)	263 (40.8%)	
Education				< 0.001
≤ high school	55 (22.8%)	110 (43.7%)	165 (32.8%)	
Some college/college	269 (77.2%)	181 (56.3%)	450 (67.2%)	
Cancer types				0.831
Breast/prostate cancer	103 (31.9%)	93 (32.8%)	196 (32.3%)	
Others	217 (68.1%)	194 (67.2%)	411 (67.7%)	
Ethnicity				0.201
Non-Hispanic white	279 (86.2%)	230 (82.2%)	509 (84.3%)	
Other	45 (13.8%)	61 (17.8%)	106 (15.7%)	
Smoked at least 100 cigarettes				< 0.001
Yes	127 (38.8%)	164 (55.5%)	291 (46.8%)	
No	197 (61.2%)	126 (44.5%)	323 (53.2%)	
Nutritional intake, mean ± SD	0.51 ± 0.19	0.55 ± 0.18	0.53 ± 0.18	0.026

The mean (sd) and proportions in the tables are weighted using the 2-year sampling weights provided by the NHANES

### Association between oral health and frailty

The associations between oral health parameters and frailty are shown in Table 2. All regression showed no multicollinearity (all VIF < 2). In the unadjusted model, all oral health parameters showed significant association with frailty. Compared with individuals reporting good health of gums and teeth, those with poor or fair health of gums and teeth were more likely to be frail (OR = 2.37, 95%CI: 1.55, 3.61, *p* < 0.001). Compared with individuals reporting never or hardly ever oral pain, oral-related depression, dietary restrictions, oral-related eating limitations, oral-related social embarrassment, and oral-related working restrictions, those who reported very often/fairly often/occasionally experiencing these conditions were more likely to be frail (ORs range from 1.92 to 7.20). In the adjusted model, 606 participants were used in the analyses. Little's MCAR test indicated that the data were missing completely at random (p = 0.716). The results demonstrated that individuals who reported worse health of gums and teeth, frequent depression because of oral conditions, dietary restrictions, oral-related eating limitations, oral-related social embarrassment, and oral-related working restrictions were identified with attenuated but significant associations with frailty (ORs range from 1.82 to 5.00). The significant associations between frailty status and oral pain was retained (OR = 2.06, 95%CI: 1.24, 3.40, *p* = 0.005). The sensitivity analysis of imputing missing data showed no substantial change in the results (Supplementary Table S4).

**Table 2:** Logistic regressions of oral health and frailty

Oral predictors	Unadjusted Model (n = 615)		Adjusted Model (n = 606)	
Health of gum and teeth	OR (95%CI)	P value	OR (95%CI)	P value
Good and above	-		-	
Poor, fair	2.37 (1.55, 3.61)	< 0.001	2.36 (1.50, 3.71)	< 0.001
Oral pain				
Never/hardly ever	-		-	
Very often/fairly often/occasionally	1.92 (1.21, 3.05)	0.006	2.06 (1.24, 3.40)	0.005
Oral related depression				
Never/hardly ever	-		-	
Very often/fairly often/occasionally	2.27 (1.28, 4.01)	0.005	1.93 (1.06, 3.49)	0.031
Dietary restriction				
Never/hardly ever	-		-	
Very often/fairly often/occasionally	2.36 (1.46, 3.81)	< 0.001	2.05 (1.24, 3.40)	0.005
Oral related eating limitation				
Never/hardly ever	-		-	
Very often/fairly often/occasionally	3.11 (1.91, 5.05)	< 0.001	2.64 (1.57, 4.44)	< 0.001
Oral related social embarrassment				
Never/hardly ever	-		-	
Very often/fairly often/occasionally	1.83 (1.10, 3.04)	0.021	1.82 (1.02, 3.24)	0.041
Oral related working restriction				
Never/hardly ever	-		-	
Very often/fairly often/occasionally	7.20 (2.10, 24.71)	0.002	5.00 (1.33, 18.80)	0.017

The adjusted model was adjusted for sex, age, education, marriage status, ethnicity, cancer type, smoking status, and nutrition index-nutrition intake

### Subgroup analyses

Subgroup analyses within the adjusted model identified significant differences between some covariate subgroups, including gender, marital status, education, and smoking status: (1) frailty risk was significantly elevated among male older cancer patients experiencing oral-related social embarrassment (OR = 5.27, 95% CI: 1.49, 18.69) but not among females (OR = 1.21, 95% CI: 0.63, 2.30); (2) frailty risk was significantly elevated among single/divorced/widowed individuals experiencing oral-related depression (OR = 4.34, 95%CI: 1.66, 11.40), dietary restrictions (OR = 3.86, 95%CI:1.76, 8.49), and eating limitations (OR = 5.25, 95%CI: 2.22, 12.42), but not among those married/living with partner; (3) frailty risk increased among less educated cancer patients experiencing oral pain (OR = 4.74, 95%CI: 1.45, 15.50), but not among individuals with higher educational levels (OR = 1.37, 95%CI: 0.75, 2.50); (4) older cancer individuals experiencing oral-related depression may have higher frailty risk (OR = 2.79, 95%CI: 1.18, 6.59), but not non-smoking individuals (OR = 0.90, 95%CI: 0.31, 3.63) (Supplementary Table S5).

## Discussion

This study has revealed an association between oral health and frailty in cancer patients. Those who reported worse health in teeth and gums, frequent oral pain, oral-related depression, dietary restrictions, oral-related eating limitations, social embarrassment, and working restrictions were more likely to suffer from frailty. Subgroup analyses suggested that males, single/divorced/widowed individuals, those with lower education levels, and smokers were at a potentially higher risk of frailty in relation to specific oral health parameters.

This study newly demonstrates the association between poor oral health and frailty in older cancer patients, extending beyond the findings in general older adult populations by highlighting a cancer-specific context. Compared with the general older population, cancer patients appeared to have higher vulnerability to frailty when reporting poor or fair health of gums and teeth (OR 2.36 vs. 1.23) [26] and frequent oral pain (OR 2.06 vs. 1.72) [27]. The association between oral health and frailty in older cancer patients may be amplified through multiple mechanisms. Nutritionally, disease progression and treatment side effects such as chemotherapy-induced nausea may intensify the impact of poor oral health on nutritional status [28]. Biologically, elevated baseline inflammation from malignancy or anticancer therapies may exacerbate the effects of oral health deterioration on muscle function and metabolism, increasing frailty risk [29]. Psychosocially, oral health–related depression, embarrassment, and activity restrictions may promote isolation, sedentary behavior, and cognitive decline, collectively contributing to frailty [30]. Furthermore, cancer-specific toxicities, such as mucositis, xerostomia, taste alterations, and infection risk, may worsen oral health, creating a bidirectional cycle wherein frailty further impairs oral hygiene maintenance [31, 41]. While previous research on frailty in older cancer patients has predominantly emphasized interventions targeting physical activity and nutrition to prevent or mitigate frailty progression [32–34], our findings highlight poor oral health as an under-explored correlate. Further prospective and interventional research is required to explore its potential role in frailty risk identification and mitigation.

This study also explored potential demographic characteristics that may modulate the association between oral health and frailty in older cancer patients. First, male cancer patients reporting oral-related social embarrassment exhibited potentially higher frailty risk than females. This may stem from males experiencing greater psychological stress from embarrassment [35], potentially amplifying frailty through stress-related pathways, such as inflammation or social withdrawal. Second, single cancer patients with frequent oral-related depression, dietary restrictions, or eating limitations may heighten frailty vulnerability. Marriage may mitigate these effects by providing social support that reduces depression and enhances psychological well-being in cancer patients [36], while alleviating dietary challenges through spousal support for healthier eating amid treatment-related barriers [37]. Third, cancer patients with lower education levels experiencing frequent oral pain may be more likely to become frail probably due to limited coping strategies and lower oral health literacy [38]. Finally, among cancer patients with oral pain, those with a significant smoking history faced elevated frailty risk, consistent with evidence linking smoking to oral tissue damage and heightened pain in cancer populations [39]. These cancer-specific demographic insights call for tailored studies to inform targeted interventions in oncology settings.

## Limitations

There are some limitations in this study. First, the cross-sectional design precludes establishing causal relationships between oral health parameters and frailty among older cancer patients. A longitudinal approach was constrained by the absence of oral health data in previous NHANES waves. Second, reliance on self-reported oral health measures and certain frailty components introduces potential recall bias. Finally, the data were collected from a public database, which was not designed to explore the association between oral health and frailty specifically in patients with cancer; consequently, some potentially relevant variables (e.g., cancer stage, cancer treatment, time since cancer diagnosis, drinking status, and physical activity) may not be adequately addressed.

## Implications

This study revealed the positive association between oral health disorders and frailty among older cancer patients. Exploratory analyses suggested that certain demographic characteristics (male sex, being single/divorced/widowed, lower education, and history of smoking) may amplify the association between oral health and frailty. Besides, healthcare providers are recommended to incorporate routine oral health assessments into the clinical management of older cancer patients. Tailored intervention programs addressing the specific needs of patient subgroups based on these demographic factors should be developed and implemented.

Future research should prioritize longitudinal designs to establish causal relationships between oral health and frailty in older cancer patients. Additionally, studies specifically examining these associations within key demographic subgroups identified in this work are warranted. Interventional studies incorporating integrated oral care programs can be conducted to determine whether targeted improvements in oral health can reduce frailty risk or severity in this population. Finally, objectively measured oral health should be considered in future studies among older cancer patients.

## Conclusion

This study demonstrated the association between poor oral health and frailty among older cancer patients. Exploratory subgroup analyses suggested that males, single/divorced/widowed individuals, those with lower education levels, and smokers may face a potentially heightened risk of frailty in relation to specific oral health parameters. Further longitudinal and interventional studies are warranted to explore the potential utility of routine oral health assessments in identifying frailty risk and informing targeted management strategies in older cancer patients.

## Supplementary Information

Below is the link to the electronic supplementary material.

Supplementary file1 (DOCX 31 KB)

## Data Availability

The datasets used and analyzed during the current study are available from the corresponding author upon reasonable request. More information about the NHANES can be obtained at: http://www.cdc. gov/nhanes.
