# Supplementary material for: Association between oral health and frailty among older cancer patients: a cross-sectional study
Source: Support Care Cancer. 2026 Apr 20;34(5):444. doi: 10.1007/s00520-026-10650-w (PMC13092524; doi:10.1007/s00520-026-10650-w)
Supplement: Supplementary file 1 — Supplementary file1 (DOCX 31 KB) [file 520_2026_10650_MOESM1_ESM.docx]

**Supplementary Table S1. Frailty index**

| **Variables** | **Scoring** |
| --- | --- |
| **Self-reported frailty index items** |  |
| Difficulty seeing even with glass | No=0, some difficulty=0.33, a lot of difficulty=0.66, cannot do at all=1 |
| Difficulty hearing even with hearing aids | No=0, some difficulty=0.33, a lot of difficulty=0.66, cannot do at all=1 |
| Difficulty walking or climbing steps | No=0, some difficulty=0.33, a lot of difficulty=0.66, cannot do at all=1 |
| Difficulty communicating | No=0, some difficulty=0.33, a lot of difficulty=0.66, cannot do at all=1 |
| Difficulty remembering or concentrating | No=0, some difficulty=0.33, a lot of difficulty=0.66, cannot do at all=1 |
| Difficulty with self-care | No=0, some difficulty=0.33, a lot of difficulty=0.66, cannot do at all=1 |
| Difficulty raising a 2-L bottle | No=0, some difficulty=0.33, a lot of difficulty=0.66, cannot do at all=1 |
| Difficulty using hands and fingers | No=0, some difficulty=0.33, a lot of difficulty=0.66, cannot do at all=1 |
| How often feel worried/nervous/anxious | A few times a year/never=0, monthly=0.5, daily/weekly=1 |
| How often feel depressed | A few times a year/never=0, monthly=0.5, daily/weekly=1 |
| With angina/angina pectoris | No=0, yes=1 |
| With heart attack | No=0, yes=1 |
| With coronary heart disease | No=0, yes=1 |
| With stroke | No=0, yes=1 |
| With thyroid condition | No=0, yes=1 |
| With arthritis | No=0, yes=1 |
| With high blood pressure | No=0, yes=1 |
| With diabetes mellitus | No=0, yes=1 |
| With congestive heart failure | No=0, yes=1 |
| With gallbladder operation | No=0, yes=1 |
| With COPD | No=0, yes=1 |
| With a liver condition | No=0, yes=1 |
| With asthma | No=0, yes=1 |
| With weak/failing kidneys | No=0, yes=1 |
| With urinary leakage | No=0, yes=1 |
| Take prescription medication, past month | No=0, yes=1 |
| General health condition (self-reported) | Excellent/very good/good=0, fair/poor=1 |
| Seen mental health professional/past year | No=0, yes=1 |
| Weight loss>5% compared with last year | No=0, yes=1 |
| **Laboratory frailty index items** |  |
| Pulse rate (bpm) | 60-90=0, other=1 |
| Systolic blood pressure (mmHg) | 90-140=0, other=1 |
| Platelet count SI (million cells/μL) | 150-450 =0, other=1 |
| Red blood cell count (million cells/μL) | M: 4.7-6.1=0, other=1; F:4.2-5.4=0, other=1 |
| Red blood distribution width (%) | 11.6-14.6=0, other=1 |
| Glycohemoglobin (%) | 0%-5.7%=0, >5.7%=1 |
| Hemoglobin (g/dL) | M: 13.5-18=0, other=1; F: 12-16=0, other=1 |
| Lymphocyte percent (%) | 20-40=0, other=1 |
| Segmented neutrophils percent (%) | 40-80=0, other=1 |

**Supplementary Table S2. Oral health questionnaire**

| **Oral health items** | **Question** | **Score** |
| --- | --- | --- |
| Health of teeth and gums | Rate the health of your teeth and gums | 1=excellent; 2=very good; 3=good; 4=faire; 5=poor |
| Oral pain | How often last year had aching in mouth | 1=never; 2=hardly ever; 3=occasionally; 4=fairly often; 5= very often |
| Oral related depression | How often felt bad because of mouth | 1=never; 2=hardly ever; 3=occasionally; 4=fairly often; 5= very often |
| Dietary restriction | Last year avoid some food because of mouth | 1=never; 2=hardly ever; 3=occasionally; 4=fairly often; 5= very often |
| Oral related eating limitation | Last year couldn’t eat because of mouth | 1=never; 2=hardly ever; 3=occasionally; 4=fairly often; 5= very often |
| Oral related social embarrassment | Last year embarrassed because of mouth | 1=never; 2=hardly ever; 3=occasionally; 4=fairly often; 5= very often |
| Oral related working restriction | Last year had difficulty working/job because of mouth | 1=never; 2=hardly ever; 3=occasionally; 4=fairly often; 5= very often |

**Supplementary Table S3. Nutritional intake**

| **Nutrient intakes** | **Score in nutritional index 0** | **Score in nutritional index 1** |
| --- | --- | --- |
|  | **Normal range** | **Abnormal range** |
| Energy (kcal/d) | M>=2400, F>=1800 | M<2400, F<1800 |
| Energy per weight (kcal/kg/d) | 25-35 | <25, >35 |
| Protein (g/d) | M>=56, F>=46 | M<56, F<46 |
| Protein per weight (g/kg/d) | <65 years, >=0.8  >=65 years, >=1 | <65 years, <0.8  >=65 years, <1 |
| Carbohydrate (g/d) | >=180 | <180 |
| Dietary fiber (g/d) | >14 | <=14 |
| Cholesterol (mg/d) | <300 | >=300 |
| Vitamin A, RAE (mcg/d) | M 900-3000, F 700-3000 | M<900 or >3000; F<700 or >3000 |
| Vitamin C (mg/d) | M 90-2000, F 75-2000 | M <90 or >2000; F <75 or >2000 |
| Vitamin K (mcg/d) | M>=120, F>=90 | M<120, F<90 |
| Thiamin (mg/d) | M>=1.2, F>=1.1 | M<1.2, F<1.1 |
| Riboflavin (mg/d) | M>=1.3, F>=1.1 | M<1.3, F<1.1 |
| Niacin (mg/d) | M 16-35, F 14-35 | M <16 or >35; F <14 or >35 |
| Folate (mcg/d) | 400-1000 | <400, >1000 |
| Calcium (mg/d) | M <=70 years, 1000-2500  M>70 years, 1200-2500  F>50 years, 1200-2500 | M<=70 years, <1000 or >2500  M>70 years, <1200 or >2500  F>50 years, <1200 or >2500 |
| Phosphorous (mg/d) | 700-4000 | <700 or >4000 |
| Magnesium (mg/d) | M>=420, F>=320 | M<420, F<320 |
| Iron (mg/d) | 8-45 | <8 or >45 |
| Zinc (mg/d) | M 11-40, F 8-40 | M <11 or >40; F <8 or >40 |
| Copper (mg/d) | 0.9-10 | <0.9 or >10 |
| Sodium (mg/d) | <=70 years, 1300-2300  >70 years, 1200-2300 | <=70 years, <1300 or >2300  >70 years, <1200 or >2300 |
| Potassium (mg/d) | >=4700 | <4700 |
| Selenium (mcg/d) | 55-400 | <55 or >400 |
| Caffeine (mg/d) | <=400 | >400 |
| Alcohol (g/d) | M<=28, F<=14 | M>28, F<14 |
| Fish oil (g/d)* | >=0.25 | <0.25 |

Note: F, female; M, male; RAE retinol activity equivalent; *fish oil is the combination between dicosahexaenoic acid (DHA) and eicosapentaenoic acid (EPA) in the nutritional intake.

**Supplementary Table S4. Sensitivity analysis using multiple imputation to handle missing data (N=615)**

| **Oral health indicators** | **OR (95%CI)** | **P value** |
| --- | --- | --- |
| Health of gum and teeth |  |  |
| Good and above | - |  |
| Poor, fair | 2.60 (2.30, 2.95) | <0.001 |
| Oral pain |  |  |
| Never/hardly ever | - |  |
| Very often/fairly often/occasionally | 1.92 (1.69, 2.20) | <0.001 |
| Oral related depression |  |  |
| Never/hardly ever | - |  |
| Very often/fairly often/occasionally | 1.95 (1.66, 2.29) | <0.001 |
| Dietary restriction |  |  |
| Never/hardly ever | - |  |
| Very often/fairly often/occasionally | 2.30 (2.00, 2.64) | <0.001 |
| Oral related eating limitation |  |  |
| Never/hardly ever | - |  |
| Very often/fairly often/occasionally | 2.52 (2.19, 2.89) | <0.001 |
| Oral related social embarrassment |  |  |
| Never/hardly ever | - |  |
| Very often/fairly often/occasionally | 2.24 (1.94, 2.60) | <0.001 |
| Oral related working restriction |  |  |
| Never/hardly ever | - |  |
| Very often/fairly often/occasionally | 4.74 (3.37, 6.67) | <0.001 |

**Supplementary Table S5. Subgroup analysis between the association of total oral health score and frailty among patients with cancer (N=606)**

|  | **Health of teeth and gum** |  | **Oral pain** |  | **Oral related depression** |  | **Dietary restriction** |  | **Eating limitation** |  | **Social embarrassment** |  | **Working restriction** |  | **Total OHQ**  **score** |  |
| --- | --- | --- | --- | --- | --- | --- | --- | --- | --- | --- | --- | --- | --- | --- | --- | --- |
|  | **OR (95%CI)** | **P for interaction** | **OR (95%CI)** | **P for interaction** | **OR (95%CI)** | **P for interaction** | **OR (95%CI)** | **P for interaction** | **OR (95%CI)** | **P for interaction** | **OR (95%CI)** | **P for interaction** | **OR (95%CI)** | **P for interaction** | **OR (95%CI)** | **P for interaction** |
| Gender |  | 0.764 |  | 0.149 |  | 0.794 |  | 0.956 |  | 0.447 |  | 0.039 |  | 0.713 |  | 0.321 |
| Male | 2.64 (1.32, 5.28) |  | 1.37 (0.62, 3.05) |  | 2.30 (0.90, 5.87) |  | 2.32 (1.00, 5.42) |  | 3.60 (1.48, 9.74) |  | 5.27 (1.49, 18.69) |  | 4.53 (0.56, 36.49) |  | 1.17 (1.08, 1.26) |  |
| Female | 2.24 (1.22, 4.11) |  | 2.93 (1.54, 5.59) |  | 1.87 (0.88, 3.99) |  | 1.99 (1.07, 3.73) |  | 2.22 (1.17, 4.23) |  | 1.21 (0.63, 2.30) |  | 5.93 (1.29, 27.34) |  | 1.11 (1.04, 1.18) |  |
| Age |  | 0.982 |  | 0.181 |  | 0.207 |  | 0.645 |  | 0.991 |  | 0.472 |  | 0.275 |  | 0.586 |
| <=70 | 2.16 (1.17, 4.01) |  | 2.85 (1.51, 5.38) |  | 2.18 (0.99, 4.80) |  | 2.08 (1.01, 4.31) |  | 2.45 (1.19, 5.04) |  | 1.42 (0.67, 3.03) |  | 7.53 (1.54, 36.89) |  | 1.13 (1.05, 1.22) |  |
| >70 | 2.27 (1.17, 4.42) |  | 1.48 (0.73, 3.03) |  | 1.14 (0.52, 2.52) |  | 1.83 (0.92, 3.63) |  | 2.61 (1.25, 5.45) |  | 2.06 (0.77, 5.49) |  | 2.27 (0.45, 11.36) |  | 1.10 (1.03, 1.18) |  |
| Marriage |  | 0.222 |  | 0.791 |  | 0.039 |  | 0.038 |  | 0.043 |  | 0.796 |  | 0.574 |  | 0.338 |
| Married/Living with partner | 1.81 (0.98, 3.35) |  | 2.14 (1.13, 4.04) |  | 1.06 (0.47, 2.38) |  | 1.26 (0.63, 2.51) |  | 1.68 (0.85, 3.31) |  | 1.69 (0.75, 3.79) |  | 3.40 (0.46, 25.15) |  | 1.01 (1.03, 1.18) |  |
| Singled/ divorced/ widowed | 3.23 (1.64, 6.36) |  | 1.86 (0.84, 4.12) |  | 4.34 (1.66, 11.40) |  | 3.86 (1.76, 8.49) |  | 5.25 (2.22, 12.42) |  | 1.84 (0.78, 4.31) |  | 7.01 (1.28, 38.48) |  | 1.16 (1.07, 1.25) |  |
| Education level |  | 0.051 |  | 0.038 |  | 0.292 |  | 0.228 |  | 0.152 |  | 0.121 |  | * |  | 0.360 |
| ≤high school | 4.69 (1.89, 11.67) |  | 4.74 (1.45, 15.50) |  | 2.69 (1.00, 7.29) |  | 3.30 (1.27, 8.58) |  | 3.92 (1.58, 9.71) |  | 1.13 (0.43, 2.94) |  | / |  | 1.17 (1.04, 1.31) |  |
| Some college/college | 1.69 (0.99, 2.99) |  | 1.37 (0.75, 2.50) |  | 1.45 (0.70, 2.98) |  | 1.61 (0.88, 2.93) |  | 1.89 (1.00, 3.57) |  | 2.76 (1.39, 5.49) |  | / |  | 1.11 (1.05, 1.17) |  |
| Cancer types |  | 0.633 |  | 0.422 |  | 0.960 |  | 0.841 |  | 0.548 |  | 0.661 |  | 0.768 |  | 0.773 |
| Breast/prostate cancer | 3.15 (1.32, 7.55) |  | 2.87 (1.19, 6.96) |  | 1.79 (0.57, 5.60) |  | 2.44 (0.92, 6.45) |  | 3.76 (1.27, 11.11) |  | 1.48 (0.38, 5.68) |  | 3.55 (0.29, 42.92) |  | 1.15 (1.02, 1.30) |  |
| Others | 2.28 (1.30, 4.00) |  | 1.93 (1.02, 3.65) |  | 2.09 (0.98, 4.42) |  | 1.94 (1.04, 3.63) |  | 2.44 (1.29, 4.62) |  | 2.12 (1.10, 4.08) |  | 6.19 (1.26, 30.28) |  | 1.13 (1.07, 1.20) |  |
| Ethnicity |  | 0.916 |  | 0.999 |  | 0.794 |  | 0.121 |  | 0.525 |  | 0.977 |  | 0.586 |  | 0.903 |
| Non-Hispanic white | 1.91 (0.73, 5.00) |  | 1.92 (0.56, 6.60) |  | 1.43 (0.52, 3.93) |  | 4.20 (1.42, 12.44) |  | 3.28 (1.16, 9.26) |  | 1.37 (0.41, 4.61) |  | 3.47 (0.73, 16.44) |  |  |  |
| Other | 2.44 (1.47, 4.06) |  | 2.08 (1.19, 3.64) |  | 2.11 (1.03, 4.31) |  | 1.69 (0.96, 2.99) |  | 2.45 (1.35, 4.46) |  | 1.95 (1.00, 3.78) |  | 7.69 (1.23, 48.28) |  |  |  |
| Smoking status |  | 0.245 |  | 0.648 |  | 0.041 |  | 0.877 |  | 0.513 |  | 0.194 |  | 0.425 |  | 0.898 |
| Yes | 3.02 (1.54, 5.91) |  | 2.10 (1.03, 4.28) |  | 2.79 (1.18, 6.59) |  | 2.07 (1.05, 4.08) |  | 2.84 (1.37, 5.86) |  | 2.40 (1.02, 5.64) |  | 3.42 (0.81, 14.55) |  | 1.12 (1.04, 1.21) |  |
| No | 1.88 (0.98, 3.59) |  | 2.02 (0.95, 4.31) |  | 0.90 (0.31, 3.63) |  | 2.00 (0.92, 4.35) |  | 2.27 (1.04, 4.99) |  | 1.38 (0.60, 3.19) |  | 13.97 (1.21, 160.57) |  | 1.13 (1.06, 1.21) |  |
| Nutritional intake |  | 0.745 |  | 0.736 |  | 0.941 |  | 0.148 |  | 0.184 |  | 0.981 |  | 0.690 |  | 0.685 |
| <= mean | 2.43 (1.24, 4.77) |  | 1.93 (0.96, 3.84) |  | 2.10 (0.82, 5.40) |  | 1.41 (0.66, 3.01) |  | 1.93 (0.89, 4.18) |  | 3.15 (0.81, 5.75) |  | 4.91 (0.36, 67.33) |  | 1.16 (1.07, 1.25) |  |
| >mean | 2.27 (1.22, 4.24) |  | 2.06 (0.98, 4.36) |  | 1.76 (0.82, 3.78) |  | 2.55 (1.31, 4.98) |  | 3.33 (1.64, 6.75) |  | 1.65 (0.80, 3.41) |  | 5.17 (1.03, 25.98) |  | 1.11 (1.04, 1.18) |  |

Notes: Adjusted for sex, age, education, marriage status, ethnicity, cancer type, smoking status, and nutritional intake; * insufficient sample in the subgroups.
